# Bacterioplankton Zonation Does Exist in High Elevation, Polymictic Lakes

**DOI:** 10.3389/fmicb.2022.764566

**Published:** 2022-02-17

**Authors:** Pablo Aguilar, Irma Vila, Ruben Sommaruga

**Affiliations:** ^1^Lake and Glacier Ecology Research Group, Department of Ecology, University of Innsbruck, Innsbruck, Austria; ^2^Laboratorio de Complejidad Microbiana y Ecología Funcional, Instituto Antofagasta, Universidad de Antofagasta, Antofagasta, Chile; ^3^Núcleo Milenio INVASAL, Concepción, Chile; ^4^Departamento de Ciencias Ecológicas, Facultad de Ciencias, Universidad de Chile, Santiago, Chile

**Keywords:** water column, 16S rRNA gene, Andean plateau, bacterioplankton diversity, polymictic lakes

## Abstract

The assessment of distribution patterns or zonation of planktonic microbes along the water column is a crucial step to interpret their function in the ecosystem. In lakes without seasonal thermal stratification or polymictic systems such as high elevation tropical lakes, planktonic bacterial taxa are probably homogeneously distributed in the water column in contrast to what is known for thermally stratified lakes. However, we know little about bacterial distribution patterns in polymictic lakes and their relation to environmental gradients other than temperature. Here we assessed the diversity, microdiversity, and bacterial community composition at different discrete depths in three high elevation lakes (4,400–4,550 m above sea level) from the Andean plateau to test whether bacterial zonation patterns exist along the water column. For this objective, we analyzed bulk DNA and the putatively active fraction (cDNA) of the 16S rRNA gene. Although a clear gradient of temperature and oxygen was not detected along the water column, a significant vertical spatial zonation of the bacterial communities was present in two out of the three lakes, with microdiversity contributing to such pattern. Our results provide a reference for understanding how changing environmental conditions could affect high elevation aquatic ecosystems, particularly when warming is amplified with elevation, accelerating changes in hydrological regimes and biodiversity. Finally, our results highlight the importance of incorporating the whole water column in ecological studies of aquatic ecosystems lacking temporal or permanent thermal stratification.

## Introduction

The assessment of distribution patterns or zonation of planktonic microbes is crucial to understand the mechanisms shaping microbial communities and their function in the ecosystem (Ladau and Eloe-Fadrosh, 2019). In lakes, spatial zonation of microbial communities along the water column has already been well documented, but mainly for deep, thermally stratified systems. For example, a clear zonation in bacterial community composition between the hypoxic/anoxic and oxygenated layers is known for several lakes around the world (Pedrós-Alió and Guerrero, 1993; Jürgens et al., 1994; Casamayor et al., 2008; Peura et al., 2012; Salmaso, 2019; Tran et al., 2021). The existence of a spatial change in the structure of a microbial community of thermally stratified waters is expected, because it is well established that microbes respond to gradients of temperature, oxygen, methane, and nutrient concentrations, among other variables (Taipale et al., 2009; Dodds et al., 2019). However, how microbial communities are spatially structured when frequent lake mixing events occur to disrupt thermal stratification or other environmental gradients temporarily is not well documented.

Most previous studies have focused on lake mixing events produced only by sporadic disturbances. For example, events such as cyclones or typhoons disrupt the thermal stratification of lakes, leading to changes in primary production (Klug et al., 2012) and merging microbial communities from the epilimnion and hypolimnion (Jones et al., 2008). Mixing events on shallow stratified lakes have also shown clear effects on the bacterial community composition and distribution (Comte et al., 2017). Deciphering the microbial spatial variability in different lake mixing types is crucial for predicting how microbial life could respond to mixing regime alterations caused by climate warming. Further, climate change is known to alter organisms dispersal and nutrient loads with environmental consequences such as shifts in productivity and food web structure (Cavicchioli et al., 2019). Such changes could be more pronounced in high-elevation lakes since warming is amplified with elevation (Pepin et al., 2015).

High elevation lakes in the Andean plateau have been described as cold polymictic with typical holomixis during the night (Hutchinson and Löffler, 1956; Mühlhauser et al., 1995; Wetzel, 2001). This means that the entire lake behaves like the epilimnion of stratified lakes that it is eventually mixed by strong wind events (Boehrer and Schultze, 2008). Current measurements of temporal mixing patterns in these polymictic lakes are missing. Further, although these lakes are characterized by a high degree of genetic novelty (Dorador et al., 2013; Filker et al., 2016), it is not well deciphered how microbial communities are spatially structured within the water column. The only study exploring bacterial communities at several depths unveiled that these lakes can sometimes be stratified, as evidenced by an anoxic water layer found at 8 m depth with high abundances of an anoxygenic phototrophic bacterium in Lake Cotacotani (Aguilar et al., 2018).

Microbial diversity in lakes has been widely explored using operational taxonomic units (OTUs), but this approach shows phylogenetic inconsistency associated with their extensive ecological heterogeneity (Koeppel and Wu, 2013). Therefore, exploring community changes across weak environmental gradients such as those found in polymictic lakes should consider the analysis of microdiversity. This includes the study of phylogenetically closely related bacterial populations composed of different geno- and phenotypes that potentially resulting functional diversity (Schloter et al., 2000). Establishing patterns of microdiversity could help to elucidate the importance of key environmental parameters governing niche differentiation that may not be identifiable at lower taxonomic resolution (Chase and Martiny, 2018). For example, microdiversity seems to explain the persistence of microbial OTUs across changing environmental conditions through the epilimnion and hypolimnion of lakes (García-García et al., 2019).

In this study, we assessed the diversity, microdiversity, and community composition of bacterioplankton along the water column of three high elevation Andean lakes with different maximum depth. We analyzed the bulk (DNA) and the putatively active (cDNA) fraction and clustered the sequences into amplicon sequence variants (ASVs) and then into OTUs at 97% similarity to assess the microdiversity. Based on the polymictic behavior described for high elevation tropical lakes (Hutchinson and Löffler, 1956), one could expect to find a weak vertical microbial zonation along the water column, but considering that apparently not all lakes in this region are holomictic (Aguilar et al., 2018) and that other environmental gradients exist (e.g., light), we hypothesized that spatial zonation of bacterioplankton does exist, especially in deep lakes. Thus, we first assessed the existence of physicochemical gradients along the water column and second, tested whether bacterial communities differ along the water column using discrete samples collected at single depths.

## Materials and Methods

### Study Area and Sampling Strategy

Sampling was done in Lake Cotacotani (hereafter, COTA; 4,550 m a.s.l.; max. depth: 20 m), Lake Chungará [hereafter, CHUN; 4,520 m a.s.l.; max. depth: 34–40 m depending on hydrological year (Mühlhauser et al., 1995)], and Lake Piacota (hereafter, PIA; 4,400 m a.s.l.; max. depth: 4 m). All lakes are located within the Lauca National Park in the Andean plateau, Northern Chile (Aguilar et al., 2018). Water samples were collected in quadruplicate (i.e., four independent samples) using a horizontal Van Dorn sampler (3 L) at 3–4 discrete depths (COTA: 0, 3, 6, and 9 m; CHUN: 0, 6, 10, and 20 m; PIA: 0, 2, and 4 m) from a boat placed over the deepest area of the lake during the wet season (April, 2017). Due to strong wind events in Lake Chungará and Lake Cotacotani, it was not possible to collect samples at deeper water layers.

Water samples were kept in cold black boxes, and afterward (within ca. 3 h) they were filtered onto 0.22 μm pore size filters (47 mm, Millipore GPWP). Filters were placed in Eppendorf tubes with RNAlater (Qiagen, Germantown, MD) and maintained at -20°C until DNA and RNA extraction took place.

### DNA and RNA Extraction and Sequencing

Three out of four samples (at every single depth) were used for RNA extraction using the RNeasy PowerWater kit (Qiagen, Germantown, MD) following the manufacturer’s protocol, followed by reverse transcription using random oligonucleotide primer and RevertAid H Minus transcriptase (Thermo Fisher, Waltham, MA, United States). The PCR of the product prior to the reverse transcription was used as negative control. Further, one sample from every single depth was used for DNA extraction using the PowerWater DNA isolation kit (Mo Bio Laboratories Inc.) following the manufacturer’s protocol. The concentration and quality of cDNA and DNA were measured with a NanoDrop spectrophotometer (NanoDrop 8,000, Thermo Scientific).

The cDNA and DNA were used as a templates for the V4−V5 region amplification of the 16S SSU rRNA with the primers 515FY (5′GTGYCAGCMGCCGCGGTAA3′) and 926R (5′CCGYCAATTYMTTTRAGTTT3′) (Parada et al., 2016). Sequencing was done at LGC Genomics (Berlin, Germany) using the Illumina MiSeq platform. Briefly, each PCR was done with 1–10 ng of DNA extract (total volume 1 μl), 15 pmol of each forward primer and reverse primer [in 20 μl volume of 1 × MyTaq buffer containing 1.5 units MyTaq DNA polymerase (Bioline)], 2 μl of BioStabII PCR Enhancer (Sigma) and additionally 0.2 μl of DNase (Articzymes). The program was set to 20 cycles, using the following parameters: 1 min 96°C predenaturation; 96°C for 15 s, 50°C for 30 s, 70°C for 90 s. For this reaction, barcoded primers 515FY/926R were added. Nucleic acids concentration of amplicons of interest was determined by gel electrophoresis. About 20 ng amplicon of each sample (*n* = 44) was pooled having different barcodes. The amplicon pools were purified with one volume AMPure XP beads (Agencourt) to remove primer dimers and other small mispriming products, followed by an additional purification step on MinElute columns (Qiagen). About 100 ng of each purified amplicon pool was used to construct Illumina libraries using the Ovation Rapid DR Multiplex System 1–96 (NuGEN). Illumina libraries were pooled and size selected by preparative gel electrophoresis. Sequencing was done on an Illumina MiSeq using V3 Chemistry (Illumina). Raw amplicon reads were deposited in the Sequence Read Archive (SRA) of NCBI under Accession no PRJNA750510.

### Amplicon Data Processing

Raw amplicons from 44 samples were analyzed using the R package DADA2, version 1.16.0 (Callahan et al., 2016). Briefly, after inspection of read quality profiles, the forward reads were trimmed to 240 bases and the reverse ones to 220 bases. All reads containing more than two expected errors were removed. The error rates were learned from a subset of 890,171 reads. These error rates were used to infer the ASVs. The forward and reverse reads were merged to obtain the full denoized sequence of ASVs. Denoized sequences with one or more mismatch in the overlap region were removed. The chimeras were removed using “removeBimeraDenovo.” Finally, ASVs were classified with the Silva reference data set version 138 using the IDTAXA algorithm (Murali et al., 2018), and a table with read counts and taxonomy of all ASVs was constructed. Sequences classified as eukaryote, archaea, chloroplast, mitochondria, and unknown kingdom were removed. Data were normalized using variance stabilizing transformation (vds/vst) in R with the package DESeq2 (Love et al., 2014).

### Statistical Analyses

The rRNA: rDNA ratio was calculated to test for differences at the phylum level between the putatively active fraction (cDNA) and the bulk fraction (DNA). Briefly, the reads associated with every phylum in each cDNA replicate (*n* = 3) were divided by the reads of the DNA fraction (*n* = 1), obtaining three rRNA: rDNA ratio values per depth which were visualized using boxplots in the package ggplot2 in R (Wickham, 2016). The putatively active fraction was used for downstream analyses. The estimates of species richness (ASVs number), Shannon and Simpson diversity indexes were calculated using the Vegan package in R (Oksanen et al., 2019). Further, phylogenetic diversity (Faith’s PD) was calculated using the Picante package in R (Kembel et al., 2010). The phylogenetic tree used for Faith’s phylogenetic diversity (Faith, 1992) was calculated using FastTree v. 2.1.11, applying the generalized time−reversible model. Dispersion analysis within each lake and ordinations (NMDS and PCoA) were performed based on Bray-Curtis distances using functions betadisper, metaMDS, and pcoa in R packages vegan and ape (Oksanen et al., 2019; Paradis and Schliep, 2019). The functional prediction of the bacterial communities was assessed using Tax4Fun2 version 1.1.5 (Wemheuer et al., 2020). To test for significant differences among samples grouped by depth in the ordinations, ANOSIM test (alpha = 0.05 and 9,999 permutations) was used.

The BIOENV function from the Vegan package in R (Oksanen et al., 2019) was used to find the subset of environmental variables that have the maximum correlation (Pearson correlation) with community dissimilarities (based on Bray-Curtis distance) within the water column of each lake.

To identify potential biotic interactions (e.g., competition or mutualism) and how niche similarity (reflected by phylogenetic relatedness) change along the water column, a network and phylogenetic structure analyses were calculated among ASVs (for cDNA only). The network analysis was calculated using CoNet implemented in Cytoscape (Peter et al., 2017). Briefly, Pearson correlation, Spearman rank correlation, mutual information, as well as Bray–Curtis and Kullback–Leibler dissimilarities were calculated for ensemble network inference. From each of these five metrics, 1,000 edges with the strongest support (for example, largest correlation coefficient) were used for threshold selection. Associations with Benjamini–Hochberg false discovery rate *p*-values > 0.05 and inconclusive sign (positive-*vs*.-negative) or with support from less than two of the five metrics were removed. The nearest taxon index (NTI) was used to determine the phylogenetic relatedness and, thus, to detect phylogenetic clustering or overdispersion. First, the function ses.mntd in the picante package was used to calculate the standardized effect size (z-score) of the MNTD (mean nearest taxon distance). Then, the NTI values were obtained multiplying the z-scores by -1. NTI values greater than +2 in a single community indicates coexisting taxa are more closely related than expected by chance (phylogenetic clustering), and NTI values less than -2 indicates coexisting taxa are more distantly related than expected by chance (phylogenetic overdispersion) (Stegen et al., 2012).

The effective microdiversity was measured according to García-García et al. (2019). First, we made a clustering of ASVs into 97% similarity OTUs using Opticlust algorithm (Westcott and Schloss, 2017) implemented in MOTHUR (Schloss et al., 2009). Then, the OTUs with a global abundance higher than 5,000 reads were selected. We calculated the global abundance of the ASV forming each OTUs selected in the previous step, followed by their rarefaction to 5,000 reads. This was done to avoid OTUs with higher global abundances being assigned a higher microdiversity (García-García et al., 2019). Further, the effective microdiversity was measured using the number of ASVs and Shannon index of the selected OTUs with the Vegan package in R (Oksanen et al., 2019). The occurrence (i.e., the fraction on samples in which the taxon had at least one read) and variability (coefficient of variation of the abundance of the taxon across the samples) of OTUs were calculated also according to García-García et al. (2019). A similarity percentages analysis (function SIMPER) was done in the Vegan package in R (Oksanen et al., 2019) to identify the contribution to the community dissimilarity by OTUs with and without microdiversity.

### Prokaryotic Abundance and Activity

Samples for determination of prokaryote abundance (10 mL) by flow cytometry were fixed with formaldehyde (final concentration 3.7%) and stored at 4°C. Prokaryote activity was assessed by the micro-centrifuge method (Kirchman, 2001) with some modifications. Briefly, ^3^H-leucine (40 μM final conc.) was added to three replicated samples (10 mL) from each single depth. Additionally, one formaldehyde-killed blank for each depth was fixed at the sampling site. ^3^H-leucine incubations were done in the dark at the *in situ* water temperature (ca. 10°C) for 30 min and they were terminated by adding formaldehyde. Samples were filtered onto white 0.22-μm polycarbonate filters (Poretics). The samples were extracted with cold trichloroacetic acid (5%) for 5 min and rinsed 3 times with the same solution. Filters were placed in scintillation vials with 5 mL of scintillation cocktail (Ready-safe, Beckman Coulter, Brea, CA, United States). The radioactivity of the filters was assessed on a scintillation counter (LS 6000IC, Beckman Coulter).

### Environmental Parameters

*In situ* vertical profiles of photosynthetically active radiation (PAR), chlorophyll-*a* (fluorescence) as a proxy for phytoplankton biomass, electrical conductivity, dissolved oxygen, pH, water temperature, and turbidity were done with a CTD Idronaut multiparameter probe (model OS316Plus). The coefficient of variation (CV) was used as a measure of relative variability of physicochemical parameters through the water column. The euphotic zone was calculated as the depth where 1% of PAR at the surface was found (Kirk, 1994). Anions (nitrate, chloride and sulfate) and cations (potassium, sodium, calcium, and magnesium) were measured by ion chromatography (Dionex ICS-1,100/1,000) at each of the different sampling depths. Further, total nitrogen and phosphorus were measured spectrophotometrically according to Mühlhauser et al. (1987). Samples at each depth were collected in pre-combusted (4 h at 450°C) glass bottles for the analysis of dissolved organic carbon (DOC) and dissolved nitrogen (DN). These samples were filtered *in situ* through pre combusted GF/F filters (Whatman). The filtrate was acidified with HCl to pH 2 and analyzed later with a Shimadzu TOC-Vc series instrument equipped with a total nitrogen module. Calibration for DOC analysis was done with potassium hydrogen phthalate, whereas for the DN, it was done with potassium nitrate. Three to five subsamples were analyzed for each sample and for a consensus reference material (CRM) for DOC (batch 5 FS-2005:0.57 mg; provided by RSMAS/MAC, University of Miami) that was run in parallel on each occasion. Results differed from the CRM given value by 5%, and the coefficient of variation among subsamples was < 2%.

## Results

### Environmental Conditions

**FIGURE 1:**
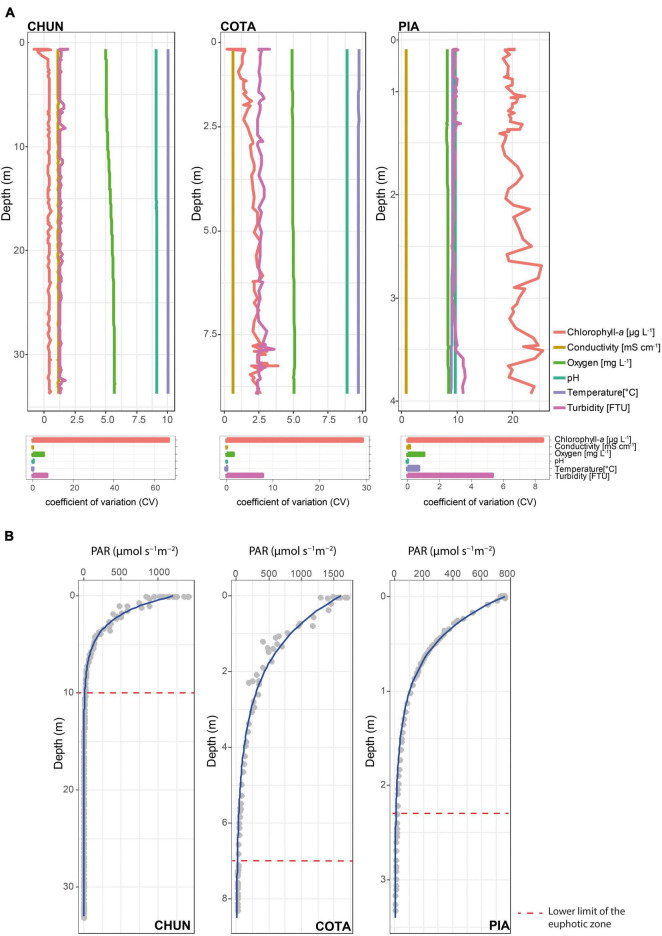
Main environmental parameters **(A)** and their coefficient of variation (CV) along the water column, and PAR values (gray circles) with the adjusted attenuation curve (blue line) **(B)** in Lake Chungará (CHUN), Lake Cotacotani (COTA), and Lake Piacota (PIA).

We did not detect pronounced gradients in electrical conductivity (CV = 0.05% in CHUN, 0.01 % in COTA, and 0.15% in PIA), pH (CV = 0.16% in CHUN, 0.02% in COTA, and 0.03% in PIA), and water temperature (CV = 0.04% in CHUN, 0.08 % in COTA, and 0.69% in PIA) as indicated by the small CV values (Figure 1A). In general for all lakes, chlorophyll-*a* showed the largest variation in the water column with the highest value found in PIA (mean = 20.6 μg L^–1^, CV = 8.46%) followed by COTA (mean = 2.06 μg L^–1^, CV = 29.1%) and CHUN (mean = 0.32 μg L^–1^, CV = 67.2%). Among the three lakes, CHUN had the largest variation in oxygen concentration (mean = 5.34 mg L^–1^, CV = 5.24%), DOC (mean = 12.36 mg L^–1^, CV = 6.6%) and dissolved nitrogen (DN; mean = 4.5 mg L^–1^, CV = 68.9%; Supplementary Table 1) along the water column. The lower limit of the euphotic zone in CHUN was located at 10 m depth, while in COTA and PIA was at 7 and 2.3 m depth, respectively (Figure 1B).

### Prokaryotic Abundance and Activity

**FIGURE 2:**
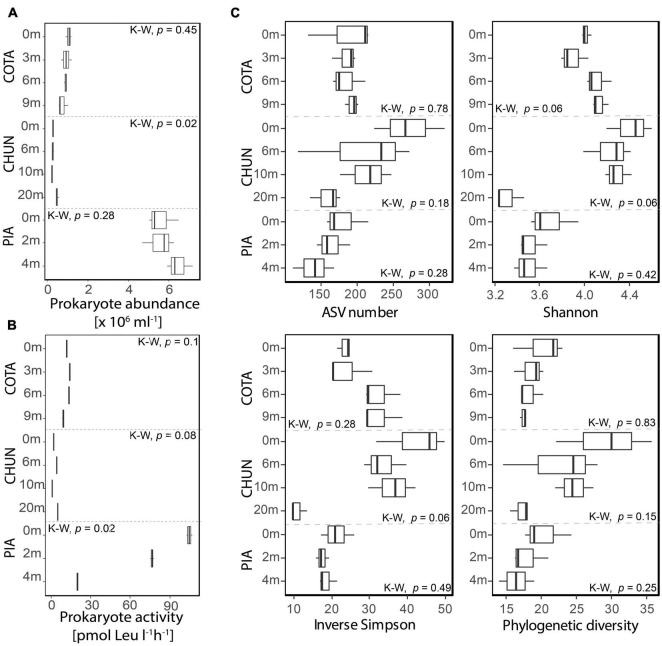
Prokaryotic abundance **(A)**, bulk ^3^H-leucine incorporation rates **(B)** and diversity metrics for the putatively active fraction **(C)** of the bacterial community from each lake. Significant differences were tested using the Kruskal–Wallis test (K-W). COTA, Lake Cotacotani; CHUN, Lake Chungará; and PIA, Lake Piacota.

The mean prokaryotic abundance in the water column of PIA (5.8 × 10^6^mL^–1^; Figure 2A) was higher than in the other two lakes (COTA: 9.1 × 10^5^mL^–1^ CHUN: 3.3 × 10^5^mL^–1^). PIA and CHUN showed the highest mean prokaryote abundance (6.4 × 10^6^mL^–1^ and 5 × 10^5^mL^–1^, respectively) at the deepest samples. However, significant differences along the water column were detected only in CHUN (Kruskal-Wallis, *p* < 0.05). The prokaryotic abundance of COTA tended to decrease with depth, but significant differences were not detected (Kruskal-Wallis, *p* > 0.05). The highest mean prokaryotic activity rates were found at the surface of PIA (104.9 pmol Leu l^–1^h^–1^) and decreased with depth (Figure 2B) showing a significant difference (Kruskal-Wallis, *p* = 0.027) among depths. Instead, significant differences along the water column were not detected in COTA (Kruskal-Wallis, COTA: *p* = 0.1) and CHUN (Kruskal-Wallis, CHUN: *p* = 0.08).

### Bacterial Community Diversity and Composition

The mean ASV number, Shannon, and Inverse Simpson for the putative active fraction tended to be higher at the surface of CHUN and PIA (Figure 2C). In contrast, the mean Shannon and inverse Simpson metrics were lower close to the surface of COTA. The phylogenetic diversity in general tended to decrease along the water column (Figure 2C), however, significant differences were not detected (Kruskal-Wallis, *p* > 0.05).

**FIGURE 3:**
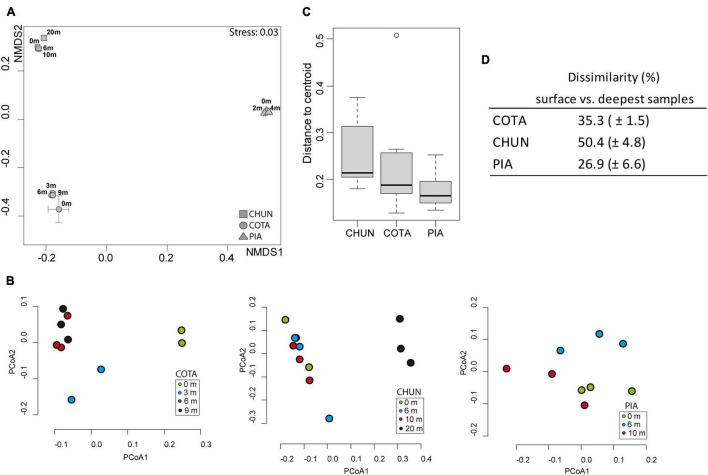
Nonmetric Multidimensional Scaling (NMDS) analysis **(A)** based on Bray–Curtis dissimilarity among lakes, Principal Coordinate Analysis (PCoA) for each lake **(B)**, Variability of communities through the water column of the lakes **(C)**, and the dissimilarity values based on Bray–Curtis between the surface and the deepest samples in each lake **(D)**. COTA, Lake Cotacotani; CHUN, Lake Chungará; and PIA, Lake Piacota.

The structure of the bacterial community corresponding to the putative active fraction was significantly different among lakes as identified by the NMDS analysis (Figure 3A) and the ANOSIM test (ANOSIM, R: 0.99, *p* < 0.001). This was true also for the composition of the bulk bacterial community composition among the lakes (ANOSIM, R: 1, *p* < 0.01). Within lakes, a clear difference among 0 m, 3 m, and the deepest samples (6 and 9 m) was observed in COTA (Figure 3B). In CHUN, the community composition at 20 m was different from the rest of the water column. There were significant differences for the putative active bacterial community only in COTA and CHUN through the water column (ANOSIM for COTA, R: 0.36, *p* < 0.001; ANOSIM for CHUN, R: 0.6, *p* = 0.0035). When the predicted metabolic functions were compared (Supplementary Figure 1), significant differences among depths were detected in CHUN (ANOSIM, R: 0.6, *p* = 0.0032) and PIA (ANOSIM, R: 0.25, *p* = 0.036) despite a clear zonation was not observed among samples. The BIOENV analysis showed that water temperature and levels of PAR had the maximum correlation (Pearson correlation = 0.98) with community dissimilarities in the water column of COTA (Supplementary Table 2); whereas in PIA, DOC, temperature and chlorophyll-*a* had the highest correlation (Pearson correlation = 0.99). In CHUN, the concentration of dissolved oxygen and DOC gave the highest correlation (Pearson correlation = 0.97). COTA and CHUN also showed the highest distance to centroids (Figure 3C) and the highest dissimilarity between the surface and the deepest samples (Figure 3D), indicating a higher variability in the water column of these two lakes compared with PIA.

**FIGURE 4:**
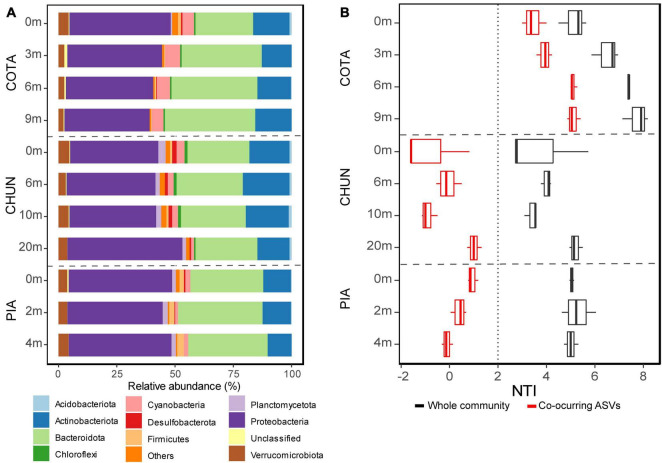
Bacterial community composition using the main phyla (>1% at least in one sample) **(A)**. The Nearest Taxon Index (NTI) indicating phylogenetic clustering (NTI > +2) of the whole community and the one considering only the pool of co-occurring ASVs identified through the network analysis **(B)**. COTA, Lake Cotacotani; CHUN, Lake Chungará; and PIA, Lake Piacota.

Proteobacteria was the most abundant phylum in all lakes and depths and represented between 36.4 and 49.5% of all sequences (Figure 4A). This phylum was followed by Bacteroidota (24.7–37% of mean relative abundance), Actinobacteriota (10.1–19.8% of mean relative abundance), and Verrucomicrobiota (2–4.4% of mean relative abundance). The putative active fraction of bacterial communities was phylogenetically clustered in all lakes (NTI > +2; Figure 4B), where such clustering increased at deeper layers in CHUN and COTA. However, when the pool of co-occurring ASVs identified through the network analysis were considered, the bacterial communities of COTA were the only presenting phylogenetic clustering. In CHUN and PIA neither phylogenetically clustering, nor overdispersion was observed.

Differences in the contribution of the different phyla to total communities was found between the bulk and the putatively active fraction (Supplementary Figure 2). For example, Cyanobacteria in COTA was the phylum with the highest rRNA:rDNA ratio (> 6.7), showing a decreasing gradient along the water column. Unclassified taxa also had a high rRNA:rDNA ratio (4.6) at the surface of the same lake. In CHUN, Cyanobacteria was the only phylum showing a high rRNA:rDNA ratio (9.7) at the surface and a low one (1.3) at 20 m depth. Instead, Acidobacteriota showed the highest rRNA:rDNA ratio (17.1), but at 6 m depth. A high number of phyla (e.g., Bacteroidota, Cyanobacteria, Proteobacteria, and Firmicutes) showed a high rRNA:rDNA ratio along the water column of PIA, with some of them (e.g., Cyanobacteria and Firmicutes) having the highest one at 4 m depth.

**FIGURE 5:**
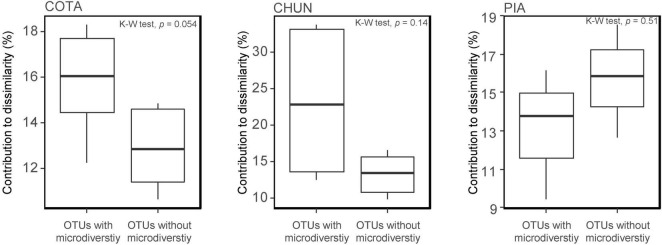
Contribution to the community dissimilarity by the most abundant OTUs (with vs. without microdiversity) in Lake Chungará (CHUN), Lake Cotacotani (COTA), and Lake Piacota (PIA).

All ASVs were clustered in 650 OTUs with only 47 OTUs detected with an abundance > 5,000 reads. A high percentage of the most abundant OTUs (COTA: 66%; CHUN: 66%; PIA: 73.6%) were present in all depths and showing little variation in their relative abundance (Supplementary Figure 3). Twenty-seven out of 47 OTUs were composed of more than one ASV (range 2–16 ASVs; Supplementary Figure 4). Further, ASVs composing a single OTU belonged generally to the same genus, but there were some exceptions such as OTU39, OTU43, OTU1, and OTU11 (Supplementary Table 3). Although significant differences in the percentage of contribution to the community dissimilarity between OTUs with and without microdiversity (a single OTU composed by more than one ASV) were not detected, microdiverse OTUs in COTA and CHUN tended to contribute more to the community dissimilarity than non-microdiverse OTUs (Figure 5). Further, the mean occurrence of microdiverse OTUs was higher only along the water column of CHUN (mean occurrence = 0.85) and COTA (mean occurrence = 0.89) compared with non-microdiverse OTUs (CHUN mean occurrence = 0.83; COTA mean occurrence = 0.73).

## Discussion

In the present study, we showed that bacterial communities along the water column changes significantly only in Lake Chungará and Lake Cotacotani, despite the lack of thermal stratification in these lakes. Actually, we expected to find a strong and clear zonation pattern in the structure of the bacterial community in the more wind-protected Lake Cotacotani where an anoxic layer was previously detected (Aguilar et al., 2018). However, the largest differences were found in the deepest system, Lake Chungará. Although this study provides the first detailed information for high elevation Andean lakes, the zonation patterns represent only a snapshot during the wet season. Given that the bacterioplankton composition changes between the dry and wet seasons (Aguilar et al., 2018), it remains to be tested how the zonation potentially develops throughout the year.

### Vertical Microbial Distribution in Polymictic Lakes

Previous studies have shown that the composition of microbial communities (based on molecular techniques) in polymictic lakes changes less throughout the water column in comparison with lakes with other mixing regimes, such as dimictic (i.e., two mixing events per year) or meromictic (i.e., no mixing) lakes. For example, the bacterial community composition in the small polymictic Lake Crystal Bog (maximum depth, 2.5 m) was not significantly different (Shade et al., 2008). Further, in Lake Xolotlán (maximum depth, 26 m), a tropical, polymictic, but low-elevation lake, bacterial production and abundance were not significantly different along the water column (Erikson et al., 1998). By contrast, the epilimnion and hypolimnion of dimictic and meromictic lakes have significantly different bacterial communities (Shade et al., 2008; Peura et al., 2012). In our study, although the bacterial community composition did not differ along the water column of Lake Piacota, we detected a significant difference in prokaryotic activity along the water column. In fact, this lake was the only one that showed a clear gradient with a high prokaryotic activity at the surface and a low one at 4 m depth (Figure 2B). Lake Chungará was the only system where prokaryotic abundance significantly changed throughout the water column. Further, we observed different vertical alpha diversity patterns in each lake, with the deepest (Chungará) and the shallowest (Piacota) lake having higher diversity at the surface than in deeper water layers. Although more lakes in other regions need to be considered, all the above mentioned variations in productivity, abundance and diversity metrics suggest that the communities in these high elevation polymictic lakes are not homogeneous with depth.

Spatial analysis of microbial communities across different ecosystems has improved the detection of microbial hotspots and the testing of ecological assembly processes (Gonzalez et al., 2012). Several variables have been identified in potentially shaping microbial communities in freshwater ecosystems such as the concentration of dissolved organic matter, pH, and hydrology (Ruiz-González et al., 2015; Niño-García et al., 2016; Muscarella et al., 2019; Ortiz-Álvarez et al., 2020). However, when the vertical dimension is considered, one of the main environmental factors determining the variability in microbial composition is the existence of a thermal stratification that creates dispersal barriers for prokaryotes and non-motile phytoplankton (Xiao et al., 2011; Peura et al., 2012; Yu et al., 2014; Cheng et al., 2020). In fact, the vertical variability of bacterioplankton in continuously or frequently mixed water bodies is not well understood, and their microbial composition cannot be compared with that of lakes with permanently or semi-permanently stratified waters, because they differ markedly in their physical, chemical and biological characteristics (Woolway and Merchant, 2019). Our hypothesis was validated since we found bacterioplankton zonation along the water column of polymictic lakes. However, such zonation was significant only in two aquatic systems, Lake Chungará and Lake Cotacotani. Although significant changes in the predicted metabolic functions were found throughout the water column of PIA, their interpretation should be done with caution since the prediction of metabolic functions has clear limitation. For example, it is not possible to detect functional variation if ASVs has identical 16S rRNA gene sequence (Langille et al., 2013). Further, the predictive performance is limited by the number of genomes available in public databases and the lack of appropriate reference data (Wemheuer et al., 2020).

Although some environmental parameters (COTA: temperature and PAR; CHUN: dissolved oxygen and DOC; PIA: DOC, temperature and chlorophyll-*a*) showed significant correlations with bacterial communities along the water column, experimental work is needed to disentangle their direct or indirect effect. Yet, we could argue that sunlight is one of the main driving factors in these high elevation ecosystems. Besides the direct effect of solar radiation (e.g., damage of the cellular machinery and inhibition of primary production) on microbial communities of high elevation lakes (Sommaruga, 2001), sunlight can indirectly affect bacteria by regulating the flux of dissolved organic matter of phytoplankton origin incorporated into biomass (Ruiz-González et al., 2013). This type of control could be particularly important in CHUN and PIA given that DOC (a proxy for DOM concentration) correlated with changes in bacterioplankton communities along the water column.

The putatively active fraction of the 16S rRNA gene represents the past and current microbial activities since this fraction is not a reliable metric for recent growth or activity (Blazewicz et al., 2013). However, its analysis in future studies would be necessary to capture the zonation of microbes with a more direct contribution to the ecosystem’s functioning than the bulk fraction. We found that Cyanobacteria showed the highest rRNA:rDNA ratio at the surface of CHUN and COTA probably having an important role for the nitrogen cycle in these nitrogen-limited ecosystems (Wurtsbaugh et al., 1985). For example, the nitrogen fixation by *Aphanizomenon*, the most abundant genus in Lake Cotacotani, releases ammonium to the surrounding water supporting primary production and the microbial food web (Adam et al., 2016).

### Microdiversity Contributes to the Microbial Zonation of High Elevation Polymictic Lakes

Ecotypes allow for fine-scale genetic diversity within an environment by occupying slightly different ecological niches. Furthermore, when they are grouped as single species they maintain their characteristic genetic signatures and ecological potential (Rocap et al., 2003; Konstantinidis and Tiedje, 2005). For example, *Polynucleobacter* ecotypes exhibit different distribution patterns depending on a lake’s thermal regime, whereas their abundance is controlled mainly by temperature (García-García et al., 2019). Thermal adaptation has also been observed in six strains with identical 16S rRNA gene sequences belonging to the Actinobacteria cluster *Luna2*, showing significant differences in their temperature curves depending on the geographic zones from which they were isolated (Hahn and Pöckl, 2005). Here, we observed that microdiverse OTUs contributed to the microbial zonation. For instance, 12 out of 14 ASVs forming the most abundant OTU (*Limnohabitans* sp.) were present in Lake Cotacotani, where six were ubiquitous throughout the water column and the other six were unique in a certain depth. However, we were not able to identify whether such zonation patterns were due to environmental factors or to other ecological processes.

*Limnohabitans* sp. (Comamonadaceae) and hgcI_clade (Sporichthyaceae) were the most abundant genera retrieved in this study, confirming our previous finding that these groups are core representatives of Andean lakes (Aguilar et al., 2018). In fact, they are commonly described as the dominant bacterial groups in freshwater environments (Newton et al., 2011). In our study, 14 ASVs classified as *Limnohabitans* sp. were grouped into only one OTU (i.e., high microdiversity), but the ASVs corresponding to the hgcI clade were grouped into six different OTUs (i.e., low microdiversity). The microdiversity of the hgcl_clade, however, could be underestimated because the 16S rRNA gene is an inefficient taxonomic marker gene for discriminating isolates at the strain level within this clade (Neuenschwander et al., 2018). The 16S rRNA gene amplicon (partial and complete sequences) has been used to assess microdiversity (Eren et al., 2013; Needham et al., 2017; Chafee et al., 2018; García-García et al., 2019; Props and Denef, 2020) and bioinformatic tools such as dada2 (Callahan et al., 2016) have been helpful maximizing the resolution of high-throughput sequencing studies that can then be used to formulate and test ecological hypotheses (Berry et al., 2017). Thus, the use of ASVs could better represent ecotypes than OTUs. However, lately it has been demonstrated that using ASVs could artificially split bacterial genomes into separate clusters producing conflicting inferences about the ecology of different ASVs from the same genome (Schloss, 2021). In this context, we suggest that future studies testing the vertical microbial zonation should include other molecular techniques such as metagenomics. This technique provides a more direct overview of the microbial metabolic role involved in biogeochemical processes along the water column of freshwater lakes (Tran et al., 2021).

## Conclusion

The analysis of bacterial communities at different depths provided an excellent opportunity to demonstrate significant differences in the zonation of microbial communities in polymictic lakes located at high elevation. Our results though limited to just one season are relevant considering that knowledge about the composition of microbial communities throughout the water column is crucial to understand local diversity and pinpoint the ecological processes that are most relevant in these remote aquatic ecosystems. Furthermore, this study provides a reference for understanding how changing environmental conditions could affect the biodiversity of these ecosystems in the short- and long-term. Finally, our results support and highlight the presence of a microbial zonation in high elevation tropical polymictic lakes.

## Data Availability Statement

The datasets presented in the study are publicly available. These data are available in the Sequence Read Archive (SRA) of NCBI under accession no. PRJNA750510.

## Author Contributions

PA collected the samples, prepared the samples for Illumina sequencing, and ran the experimental and bioinformatic analysis. PA and RS wrote most of the manuscript. IV contributed with the writing. IV and RS obtained funding for the project. All authors have read and approved this manuscript.

## Conflict of Interest

The authors declare that the research was conducted in the absence of any commercial or financial relationships that could be construed as a potential conflict of interest.

## Publisher’s Note

All claims expressed in this article are solely those of the authors and do not necessarily represent those of their affiliated organizations, or those of the publisher, the editors and the reviewers. Any product that may be evaluated in this article, or claim that may be made by its manufacturer, is not guaranteed or endorsed by the publisher.
