# Supplementary material for: Bacterioplankton Zonation Does Exist in High Elevation, Polymictic Lakes
Source: Front Microbiol. 2022 Feb 17;13:764566. doi: 10.3389/fmicb.2022.764566 (PMC8891803; doi:10.3389/fmicb.2022.764566)

Supplementary material

Bacterioplankton zonation does exist in high elevation, polymictic lakes

Pablo Aguilar[1,2,3], Irma Vila[3,4], and Ruben Sommaruga[1]

[1]Lake and Glacier Ecology Research Group, Department of Ecology, University of Innsbruck, Austria.

[2]Laboratorio de Complejidad Microbiana y Ecología Funcional, Instituto Antofagasta, Universidad de Antofagasta, Chile.

[3]Núcleo Milenio INVASAL, Concepción 3349001, Chile.

[4]Departamento de Ciencias Ecológicas, Facultad de Ciencias, Universidad de Chile, Santiago, Chile.

Correspondence: Pablo Aguilar, Laboratorio de Complejidad Microbiana y Ecología Funcional, Instituto Antofagasta, Universidad de Antofagasta, Chile. pablo.aguilar@uantof.cl

Running title: Bacterioplankton zonation in polymictic lakes

Supplementary Table 1. Environmental parameters at different depth in Lake Cotacotani (COTA), Lake Chungará (CHUN), and Lake Piacota (PIA).

	COTA				CHUN				PIA		
	**0m**	**3m**	**6m**	**9m**	**0m**	**6m**	**10m**	**20m**	**0m**	**2m**	**4m**
**Cl^- [mg L^{-1}]**	35.45	NA	NA	28.36	63.81	NA	63.81	63.81	63.81	NA	56.72
**SO_4^{2-} [mg L^{-1}]**	17.45	NA	NA	18.63	16.89	NA	16.89	16.82	19.58	NA	21.39
**Na^+ [mg L^{-1}]**	153.68	NA	NA	157.85	201.33	NA	204.13	203.32	195.30	NA	198.47
**K^+ [mg L^{-1}]**	13.96	NA	NA	14.33	36.91	NA	37.58	37.66	34.27	NA	35.96
**Mg^{2+} [mg L^{-1}]**	274.13	NA	NA	273.71	480.54	NA	482.19	476.61	300.39	NA	332.24
**Ca^{2+} [mg L^{-1}]**	14.83	NA	NA	14.54	15.57	NA	15.28	14.74	8.58	NA	8.91
**Total nitrogen [mg L^{-1}]**	0.55	NA	NA	0.43	0.34	NA	0.24	0.25	0.58	NA	0.68
**Total phosphorus [mg L^{-1}]**	1.27	NA	NA	1.16	1.91	NA	1.82	1.74	0.61	NA	0.68
**NH_4^+[mg L^{-1}]**	0.02	NA	NA	0.02	0.05	NA	0.04	0.04	0.02	NA	0.02
**NO_2^-[mg L^{-1}]**	0.0005	NA	NA	0.0005	0.0005	NA	0.0005	0.0005	0.0005	NA	0.0005
**NO_3^-[mg L^{-1}]**	0.0001	NA	NA	0.0001	0.0001	NA	0.0001	0.0001	0.0028	NA	0.0002
**N-Kjeldahl[mg L^{-1}]**	0.53	NA	NA	0.42	0.29	NA	0.20	0.21	0.57	NA	0.67
**P-Kjeldahl[mg L^{-1}]**	0.49	NA	NA	0.37	0.40	NA	0.26	0.20	0.58	NA	0.64
**PO_4^{3-}[mg L^{-1}]**	0.78	NA	NA	0.79	1.51	NA	1.56	1.54	0.03	NA	0.04
**DOC [mg L^{-1}]**	6.33	6.90	6.80	5.85	12.15	11.80	11.90	13.60	22.50	23.90	22.80
**DN [mg L^{-1}]**	2.00	1.34	2.22	0.60	8.42	0.99	3.52	4.99	1.60	1.62	1.71

Total phosphorus corresponds to phosphates and P-kjeldahl
Total nitrogen corresponds to nitrite, nitrate, ammonium and N-kjeldahl
NA : data not available

Supplementary Table 2. Subset of environmental variables with best Pearson correlation to community data in Lake Chungará (CHUN), Lake Cotacotani (COTA), and Lake Piacota (PIA) using the BIOENV function in the package Vegan. CHL: chlorophyll-a. T: temperature. PAR: photosynthetically active radiation. OXY: oxygen. DOC: dissolved organic carbon. DN: dissolved nitrogen. TURB: turbidity. COND: coductivity

	size	Pearson correlation
**COTA**		
CHL	1	0.9483
TEMP PAR	2	0.9831
TEMP CHL PAR	3	0.9809
TEMP OXY CHL PAR	4	0.9696
pH TEMP OXY CHL PAR	5	0.9346
pH TEMP OXY CHL TURB PAR	6	0.8813
pH DN TEMP OXY CHL TURB PAR	7	0.7221
pH DOC DN TEMP OXY CHL TURB PAR	8	0.5046
Best model has 2 parameters (max. 8 allowed) :		
**TEMP PAR**		
with correlation 0.9831288		

	size	Pearson correlation
**CHUN**		
DOC	1	0.9727
DOC OXY	2	0.9756
DOC OXY PAR	3	0.9496
DOC DN TEMP OXY	4	0.8927
DOC DN TEMP OXY PAR	5	0.9208
pH DOC TEMP OXY TURB PAR	6	0.8306
pH DOC DN TEMP COND OXY PAR	7	0.7022
pH DOC DN TEMP COND OXY TURB PAR	8	0.5388
pH DOC DN TEMP COND OXY CHL TURB PAR	9	0.361
Best model has 2 parameters (max. 9 allowed):		
**DOC OXY**		
with correlation 0.9756082		

	size	Pearson correlation
**PIA**		
CHL	1	0.938
CHL TURB	2	0.9087
DOC TEMP CHL	3	0.9959
pH DOC CHL TURB	4	0.9907
DOC DN TEMP CHL TURB	5	0.8911
DOC DN TEMP COND CHL TURB	6	0.8363
DOC DN TEMP COND OXY CHL TURB	7	0.7771
DOC DN TEMP COND OXY CHL TURB PAR	8	0.7271
pH DOC DN TEMP COND OXY CHL TURB PAR	9	0.6107
Best model has 3 parameters (max. 9 allowed):		
**DOC TEMP CHL**		
with correlation 0.9959058		

Supplementary Table 3. Taxonomical classification (based on Silva database version 138) for the ASVs conforming each OTUs with microdiversity.

	Phylum	Class	Order	Family	Genus
**OTU019**					
ASV34	Bacteroidota	Bacteroidia	Flavobacteriales	Flavobacteriaceae	Flavobacterium
ASV65	Bacteroidota	Bacteroidia	Flavobacteriales	Flavobacteriaceae	Flavobacterium
ASV39	Bacteroidota	Bacteroidia	Flavobacteriales	Flavobacteriaceae	Flavobacterium
ASV608	Bacteroidota	Bacteroidia	Flavobacteriales	Flavobacteriaceae	Flavobacterium
ASV937	Bacteroidota	Bacteroidia	Flavobacteriales	Flavobacteriaceae	Flavobacterium
ASV471	Bacteroidota	Bacteroidia	Flavobacteriales	Flavobacteriaceae	Flavobacterium
ASV531	Bacteroidota	Bacteroidia	Flavobacteriales	Flavobacteriaceae	Flavobacterium
ASV358	Bacteroidota	Bacteroidia	Flavobacteriales	Flavobacteriaceae	Flavobacterium
ASV86	Bacteroidota	Bacteroidia	Flavobacteriales	Flavobacteriaceae	Flavobacterium
ASV453	Bacteroidota	Bacteroidia	Flavobacteriales	Flavobacteriaceae	Flavobacterium
ASV116	Bacteroidota	Bacteroidia	Flavobacteriales	Flavobacteriaceae	Flavobacterium
ASV308	Bacteroidota	Bacteroidia	Flavobacteriales	Flavobacteriaceae	Flavobacterium
ASV115	Bacteroidota	Bacteroidia	Flavobacteriales	Flavobacteriaceae	Flavobacterium
**OTU039**					
ASV147	Actinobacteriota	Actinobacteria	Frankiales	Sporichthyaceae	hgcI_clade
ASV439	Actinobacteriota	Actinobacteria	Frankiales	Sporichthyaceae	Candidatus_Planktophila
ASV638	Actinobacteriota	Actinobacteria	Frankiales	Sporichthyaceae	hgcI_clade
ASV511	Actinobacteriota	Actinobacteria	Frankiales	Sporichthyaceae	hgcI_clade
ASV728	Actinobacteriota	Actinobacteria	Frankiales	Sporichthyaceae	hgcI_clade
ASV215	Actinobacteriota	Actinobacteria	Frankiales	Sporichthyaceae	hgcI_clade
ASV838	Actinobacteriota	Actinobacteria	Frankiales	Sporichthyaceae	Candidatus_Planktophila
ASV256	Actinobacteriota	Actinobacteria	Frankiales	Sporichthyaceae	Unclassified
ASV79	Actinobacteriota	Actinobacteria	Frankiales	Sporichthyaceae	hgcI_clade
ASV676	Actinobacteriota	Actinobacteria	Frankiales	Sporichthyaceae	Unclassified
ASV202	Actinobacteriota	Actinobacteria	Frankiales	Sporichthyaceae	Candidatus_Planktophila
ASV285	Actinobacteriota	Actinobacteria	Frankiales	Sporichthyaceae	Candidatus_Planktophila
ASV31	Actinobacteriota	Actinobacteria	Frankiales	Sporichthyaceae	Candidatus_Planktophila
**OTU038**					
ASV102	Bacteroidota	Bacteroidia	Flavobacteriales	Flavobacteriaceae	Flavobacterium
ASV318	Bacteroidota	Bacteroidia	Flavobacteriales	Flavobacteriaceae	Flavobacterium
ASV204	Bacteroidota	Bacteroidia	Flavobacteriales	Flavobacteriaceae	Flavobacterium
ASV201	Bacteroidota	Bacteroidia	Flavobacteriales	Flavobacteriaceae	Flavobacterium
ASV269	Bacteroidota	Bacteroidia	Flavobacteriales	Flavobacteriaceae	Flavobacterium
ASV97	Bacteroidota	Bacteroidia	Flavobacteriales	Flavobacteriaceae	Flavobacterium
ASV620	Bacteroidota	Bacteroidia	Flavobacteriales	Flavobacteriaceae	Flavobacterium
**OTU018**					
ASV283	Bacteroidota	Bacteroidia	Flavobacteriales	Crocinitomicaceae	Fluviicola
ASV664	Bacteroidota	Bacteroidia	Flavobacteriales	Crocinitomicaceae	Fluviicola
ASV91	Bacteroidota	Bacteroidia	Flavobacteriales	Crocinitomicaceae	Fluviicola
ASV144	Bacteroidota	Bacteroidia	Flavobacteriales	Crocinitomicaceae	Fluviicola
ASV437	Bacteroidota	Bacteroidia	Flavobacteriales	Crocinitomicaceae	Fluviicola
ASV712	Bacteroidota	Bacteroidia	Flavobacteriales	Crocinitomicaceae	Fluviicola
ASV343	Bacteroidota	Bacteroidia	Flavobacteriales	Crocinitomicaceae	Fluviicola
ASV59	Bacteroidota	Bacteroidia	Flavobacteriales	Crocinitomicaceae	Fluviicola
ASV239	Bacteroidota	Bacteroidia	Flavobacteriales	Crocinitomicaceae	Fluviicola
ASV133	Bacteroidota	Bacteroidia	Flavobacteriales	Crocinitomicaceae	Fluviicola
**OTU012**					
ASV17	Bacteroidota	Bacteroidia	Flavobacteriales	Crocinitomicaceae	Fluviicola
ASV124	Bacteroidota	Bacteroidia	Flavobacteriales	Crocinitomicaceae	Fluviicola
ASV242	Bacteroidota	Bacteroidia	Flavobacteriales	Crocinitomicaceae	Fluviicola
ASV238	Bacteroidota	Bacteroidia	Flavobacteriales	Crocinitomicaceae	Fluviicola
ASV182	Bacteroidota	Bacteroidia	Flavobacteriales	Crocinitomicaceae	Fluviicola
ASV95	Bacteroidota	Bacteroidia	Flavobacteriales	Crocinitomicaceae	Fluviicola
ASV213	Bacteroidota	Bacteroidia	Flavobacteriales	Crocinitomicaceae	Fluviicola
ASV282	Bacteroidota	Bacteroidia	Flavobacteriales	Crocinitomicaceae	Fluviicola
**OTU043**					

ASV104	Proteobacteria	Alphaproteobacteria	Rhodobacterales	Rhodobacteraceae	Rhodobacter
ASV330	Proteobacteria	Alphaproteobacteria	Rhodobacterales	Rhodobacteraceae	Rhodobacter
ASV271	Proteobacteria	Alphaproteobacteria	Rhodobacterales	Rhodobacteraceae	Gemmobacter
ASV588	Proteobacteria	Alphaproteobacteria	Rhodobacterales	Rhodobacteraceae	Rhodobacter
ASV769	Proteobacteria	Alphaproteobacteria	Rhodobacterales	Rhodobacteraceae	Pseudorhodobacter
ASV145	Proteobacteria	Alphaproteobacteria	Rhodobacterales	Rhodobacteraceae	Pseudorhodobacter
ASV360	Proteobacteria	Alphaproteobacteria	Rhodobacterales	Rhodobacteraceae	Rhodobacter
ASV753	Proteobacteria	Alphaproteobacteria	Rhodobacterales	Rhodobacteraceae	Pseudorhodobacter
ASV324	Proteobacteria	Alphaproteobacteria	Rhodobacterales	Rhodobacteraceae	Rhodobacter
ASV655	Proteobacteria	Alphaproteobacteria	Rhodobacterales	Rhodobacteraceae	Tabrizicola
ASV490	Proteobacteria	Alphaproteobacteria	Rhodobacterales	Rhodobacteraceae	Pseudorhodobacter
ASV792	Proteobacteria	Alphaproteobacteria	Rhodobacterales	Rhodobacteraceae	Pseudorhodobacter
ASV304	Proteobacteria	Alphaproteobacteria	Rhodobacterales	Rhodobacteraceae	Rhodobacter
ASV418	Proteobacteria	Alphaproteobacteria	Rhodobacterales	Rhodobacteraceae	Rhodobacter
ASV499	Proteobacteria	Alphaproteobacteria	Rhodobacterales	Rhodobacteraceae	Pseudorhodobacter
ASV803	Proteobacteria	Alphaproteobacteria	Rhodobacterales	Rhodobacteraceae	Pseudorhodobacter
**OTU001**					
ASV126	Proteobacteria	Gammaproteobacteria	Burkholderiales	Comamonadaceae	Unclassified
ASV127	Proteobacteria	Gammaproteobacteria	Burkholderiales	Comamonadaceae	Rhodoferax
ASV184	Proteobacteria	Gammaproteobacteria	Burkholderiales	Comamonadaceae	Ramlibacter
ASV650	Proteobacteria	Gammaproteobacteria	Burkholderiales	Comamonadaceae	Unclassified
ASV731	Proteobacteria	Gammaproteobacteria	Burkholderiales	Comamonadaceae	Rhodoferax
ASV874	Proteobacteria	Gammaproteobacteria	Burkholderiales	Comamonadaceae	Unclassified
ASV1	Proteobacteria	Gammaproteobacteria	Burkholderiales	Comamonadaceae	Limnohabitans
ASV510	Proteobacteria	Gammaproteobacteria	Burkholderiales	Comamonadaceae	Limnohabitans
ASV871	Proteobacteria	Gammaproteobacteria	Burkholderiales	Comamonadaceae	Rhodoferax
ASV89	Proteobacteria	Gammaproteobacteria	Burkholderiales	Comamonadaceae	Unclassified
ASV237	Proteobacteria	Gammaproteobacteria	Burkholderiales	Comamonadaceae	Rhodoferax
ASV359	Proteobacteria	Gammaproteobacteria	Burkholderiales	Comamonadaceae	Unclassified
ASV60	Proteobacteria	Gammaproteobacteria	Burkholderiales	Comamonadaceae	Unclassified
ASV3	Proteobacteria	Gammaproteobacteria	Burkholderiales	Comamonadaceae	Limnohabitans
**OTU036**					
ASV103	Bacteroidota	Bacteroidia	Cytophagales	Spirosomaceae	Pseudarcicella
ASV55	Bacteroidota	Bacteroidia	Cytophagales	Spirosomaceae	Pseudarcicella
ASV180	Bacteroidota	Bacteroidia	Cytophagales	Spirosomaceae	Pseudarcicella
**OTU020**					
ASV25	Actinobacteriota	Actinobacteria	Frankiales	Sporichthyaceae	hgcI_clade
ASV130	Actinobacteriota	Actinobacteria	Frankiales	Sporichthyaceae	hgcI_clade
ASV21	Actinobacteriota	Actinobacteria	Frankiales	Sporichthyaceae	hgcI_clade
**OTU011**					
ASV194	Proteobacteria	Gammaproteobacteria	Burkholderiales	Comamonadaceae	Hydrogenophaga
ASV36	Proteobacteria	Gammaproteobacteria	Burkholderiales	Comamonadaceae	Hydrogenophaga
ASV33	Proteobacteria	Gammaproteobacteria	Burkholderiales	Comamonadaceae	Hydrogenophaga
ASV403	Proteobacteria	Gammaproteobacteria	Burkholderiales	Comamonadaceae	Hydrogenophaga
ASV683	Proteobacteria	Gammaproteobacteria	Burkholderiales	Comamonadaceae	Hydrogenophaga
ASV71	Proteobacteria	Gammaproteobacteria	Burkholderiales	Comamonadaceae	Hydrogenophaga
ASV814	Proteobacteria	Gammaproteobacteria	Burkholderiales	Comamonadaceae	Hydrogenophaga
ASV675	Proteobacteria	Gammaproteobacteria	Burkholderiales	Comamonadaceae	Ramlibacter
ASV4	Proteobacteria	Gammaproteobacteria	Burkholderiales	Comamonadaceae	Hydrogenophaga
ASV800	Proteobacteria	Gammaproteobacteria	Burkholderiales	Comamonadaceae	Hydrogenophaga
ASV820	Proteobacteria	Gammaproteobacteria	Burkholderiales	Comamonadaceae	Unclassified
**OTU037**					
ASV101	Proteobacteria	Alphaproteobacteria	Sphingomonadales	Sphingomonadaceae	Sphingorhabdus
ASV13	Proteobacteria	Alphaproteobacteria	Sphingomonadales	Sphingomonadaceae	Sphingorhabdus
ASV265	Proteobacteria	Alphaproteobacteria	Sphingomonadales	Sphingomonadaceae	Sphingorhabdus
ASV156	Proteobacteria	Alphaproteobacteria	Sphingomonadales	Sphingomonadaceae	Sphingorhabdus
**OTU003**					
ASV106	Bacteroidota	Bacteroidia	Cytophagales	Cyclobacteriaceae	Algoriphagus
ASV309	Bacteroidota	Bacteroidia	Cytophagales	Cyclobacteriaceae	Algoriphagus
ASV8	Bacteroidota	Bacteroidia	Cytophagales	Cyclobacteriaceae	Algoriphagus
ASV99	Bacteroidota	Bacteroidia	Cytophagales	Cyclobacteriaceae	Algoriphagus
ASV75	Bacteroidota	Bacteroidia	Cytophagales	Cyclobacteriaceae	Algoriphagus
**OTU029**					
ASV14	Actinobacteriota	Actinobacteria	Corynebacteriales	Mycobacteriaceae	Mycobacterium

ASV82	Actinobacteriota	Actinobacteria	Corynebacteriales	Mycobacteriaceae	Mycobacterium
ASV210	Actinobacteriota	Actinobacteria	Corynebacteriales	Mycobacteriaceae	Mycobacterium
**OTU021**					
ASV254	Proteobacteria	Gammaproteobacteria	Cellvibrionales	Cellvibrionaceae	Cellvibrio
ASV69	Proteobacteria	Gammaproteobacteria	Cellvibrionales	Cellvibrionaceae	Cellvibrio
ASV85	Proteobacteria	Gammaproteobacteria	Cellvibrionales	Cellvibrionaceae	Cellvibrio
ASV627	Proteobacteria	Gammaproteobacteria	Cellvibrionales	Cellvibrionaceae	Cellvibrio
**OTU023**					
ASV430	Bacteroidota	Bacteroidia	Flavobacteriales	Cryomorphaceae	Unclassified
ASV47	Bacteroidota	Bacteroidia	Flavobacteriales	Cryomorphaceae	Unclassified
ASV92	Bacteroidota	Bacteroidia	Flavobacteriales	Cryomorphaceae	Unclassified
ASV262	Bacteroidota	Bacteroidia	Flavobacteriales	Cryomorphaceae	Unclassified
**OTU006**					
ASV57	Cyanobacteria	Cyanobacteriia	Cyanobacteriales	Nostocaceae	Aphanizomenon_NIES81
ASV415	Cyanobacteria	Cyanobacteriia	Cyanobacteriales	Nostocaceae	Aphanizomenon_NIES81
ASV67	Cyanobacteria	Cyanobacteriia	Cyanobacteriales	Nostocaceae	Aphanizomenon_NIES81
**OTU027**					
ASV187	Bacteroidota	Bacteroidia	Flavobacteriales	Crocinitomicaceae	Fluviicola
ASV22	Bacteroidota	Bacteroidia	Flavobacteriales	Crocinitomicaceae	Fluviicola
ASV157	Bacteroidota	Bacteroidia	Flavobacteriales	Crocinitomicaceae	Fluviicola
**OTU008**					
ASV7	Proteobacteria	Gammaproteobacteria	Burkholderiales	Burkholderiaceae	Polynucleobacter
ASV37	Proteobacteria	Gammaproteobacteria	Burkholderiales	Burkholderiaceae	Polynucleobacter
**OTU041**					
ASV150	Proteobacteria	Alphaproteobacteria	Caulobacterales	Caulobacteraceae	Caulobacter
ASV371	Proteobacteria	Alphaproteobacteria	Caulobacterales	Caulobacteraceae	Caulobacter
ASV629	Proteobacteria	Alphaproteobacteria	Caulobacterales	Caulobacteraceae	Caulobacter
ASV266	Proteobacteria	Alphaproteobacteria	Caulobacterales	Caulobacteraceae	Caulobacter
ASV368	Proteobacteria	Alphaproteobacteria	Caulobacterales	Caulobacteraceae	Caulobacter
**OTU042**					
ASV18	Proteobacteria	Gammaproteobacteria	Alteromonadales	Alteromonadaceae	Rheinheimera
ASV220	Proteobacteria	Gammaproteobacteria	Alteromonadales	Alteromonadaceae	Rheinheimera
ASV654	Proteobacteria	Gammaproteobacteria	Alteromonadales	Alteromonadaceae	Rheinheimera
**OTU044**					
ASV11	Proteobacteria	Gammaproteobacteria	Burkholderiales	Alcaligenaceae	GKS98_freshwater_group
ASV477	Proteobacteria	Gammaproteobacteria	Burkholderiales	Alcaligenaceae	Unclassified
ASV421	Proteobacteria	Gammaproteobacteria	Burkholderiales	Alcaligenaceae	GKS98_freshwater_group
**OTU046**					
ASV93	Proteobacteria	Gammaproteobacteria	Burkholderiales	Burkholderiaceae	Lautropia
ASV310	Proteobacteria	Gammaproteobacteria	Burkholderiales	Burkholderiaceae	Lautropia
**OTU026**					
ASV114	Proteobacteria	Alphaproteobacteria	Sphingomonadales	Sphingomonadaceae	Sphingobium
ASV516	Proteobacteria	Alphaproteobacteria	Sphingomonadales	Sphingomonadaceae	Sphingobium
**OTU034**					
ASV23	Verrucomicrobiota	Verrucomicrobiae	Unclassified	Unclassified	Unclassified
ASV474	Verrucomicrobiota	Verrucomicrobiae	Unclassified	Unclassified	Unclassified
**OTU035**					
ASV77	Acidobacteriota	Acidobacteriae	Acidobacteriae_or	Acidobacteriae_fa	Paludibaculum
ASV601	Acidobacteriota	Acidobacteriae	Acidobacteriae_or	Acidobacteriae_fa	Paludibaculum
**OTU024**					
ASV822	Cyanobacteria	Cyanobacteriia	Synechococcales	Cyanobiaceae	Cyanobium_PCC-6307
ASV468	Cyanobacteria	Cyanobacteriia	Synechococcales	Cyanobiaceae	Cyanobium_PCC-6307
ASV29	Cyanobacteria	Cyanobacteriia	Synechococcales	Cyanobiaceae	Cyanobium_PCC-6307
**OTU017**					
ASV80	Bacteroidota	Bacteroidia	Cytophagales	Cyclobacteriaceae	Unclassified
ASV560	Bacteroidota	Bacteroidia	Cytophagales	Cyclobacteriaceae	Unclassified

Supplementary Figure 1. NMDS analysis using the predicted metabolic functions obtained by TAX4FUN2 in Lake Chungará (CHUN), Lake Cotacotani (COTA), and Lake Piacota (PIA).

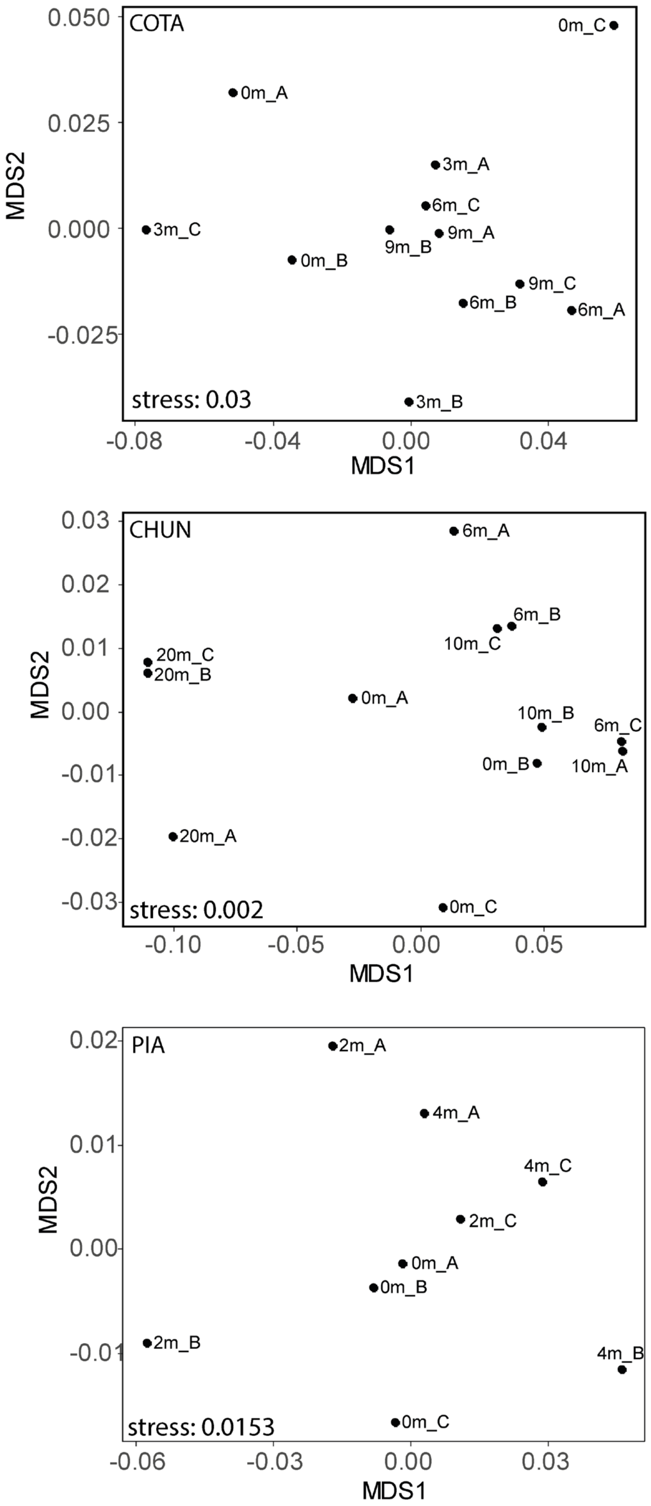

Supplementary Figure 2. Boxplots of the ratios of 16S rRNA to 16S rDNA are shown for major taxonomic groups in Lake Chungará (CHUN), Lake Cotacotani (COTA), and Lake Piacota (PIA).

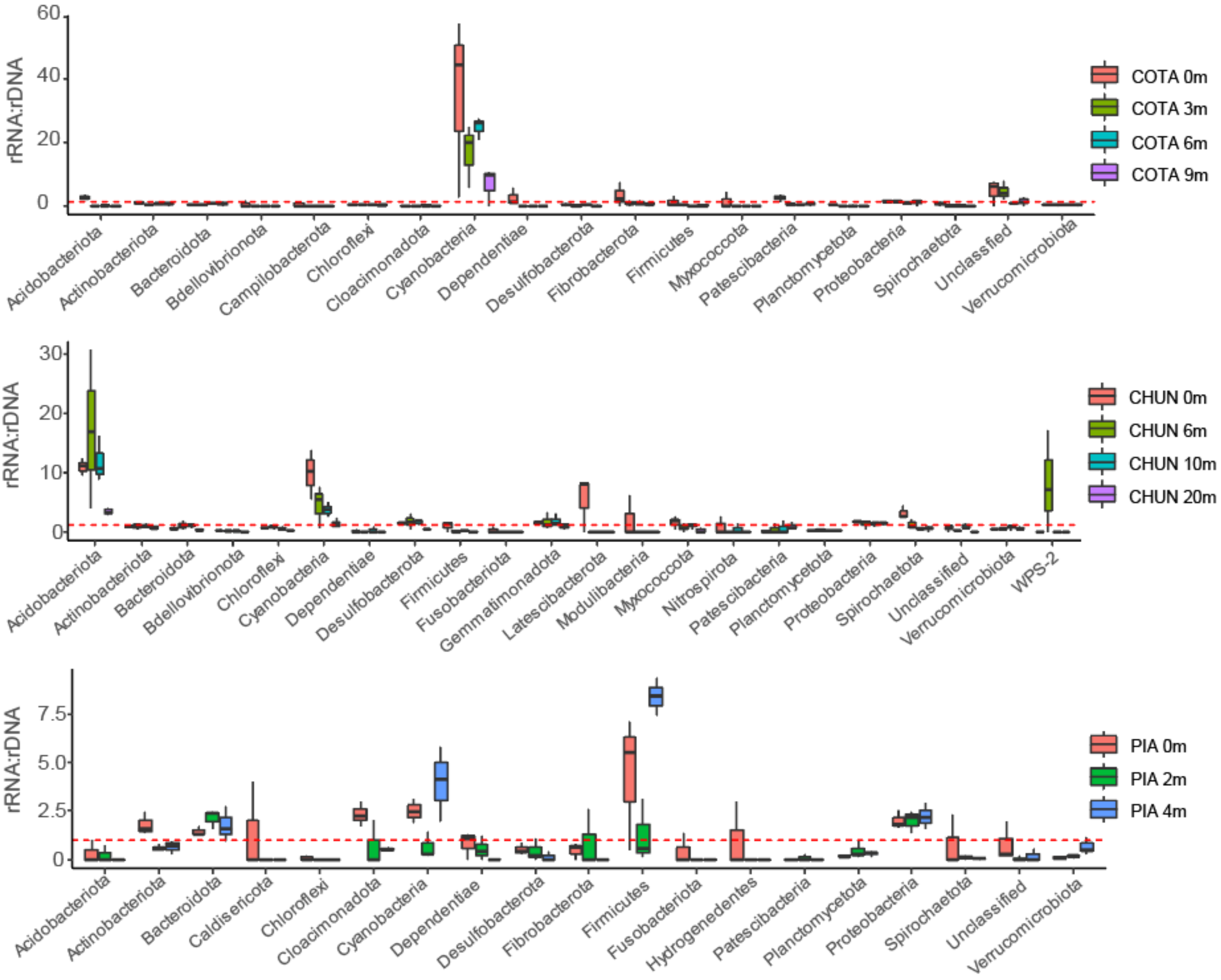

Supplementary Figure 3. Bubble plots indicating the occurrence and variability of OTUs present in all depths of Lake Chungará (CHUN), Lake Cotacotani (COTA), and Lake Piacota (PIA) considering their abundance (reads number).

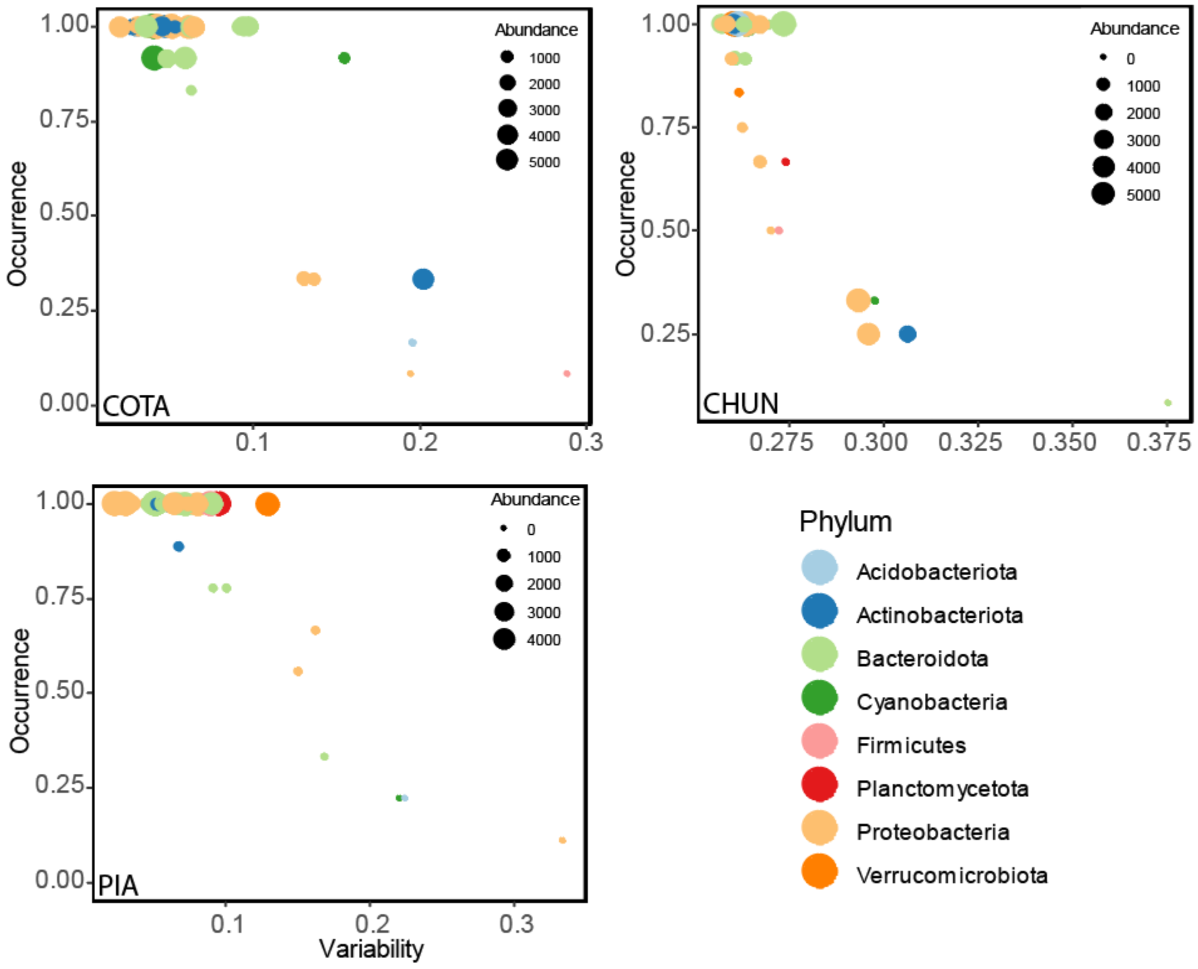

Supplementary Figure 4. Number of ASVs contained within each of the 47 most abundant OTUs (>5000 reads) and their Shannon index. The taxonomic classification at the genus level is indicated for each OTU on the X axis.

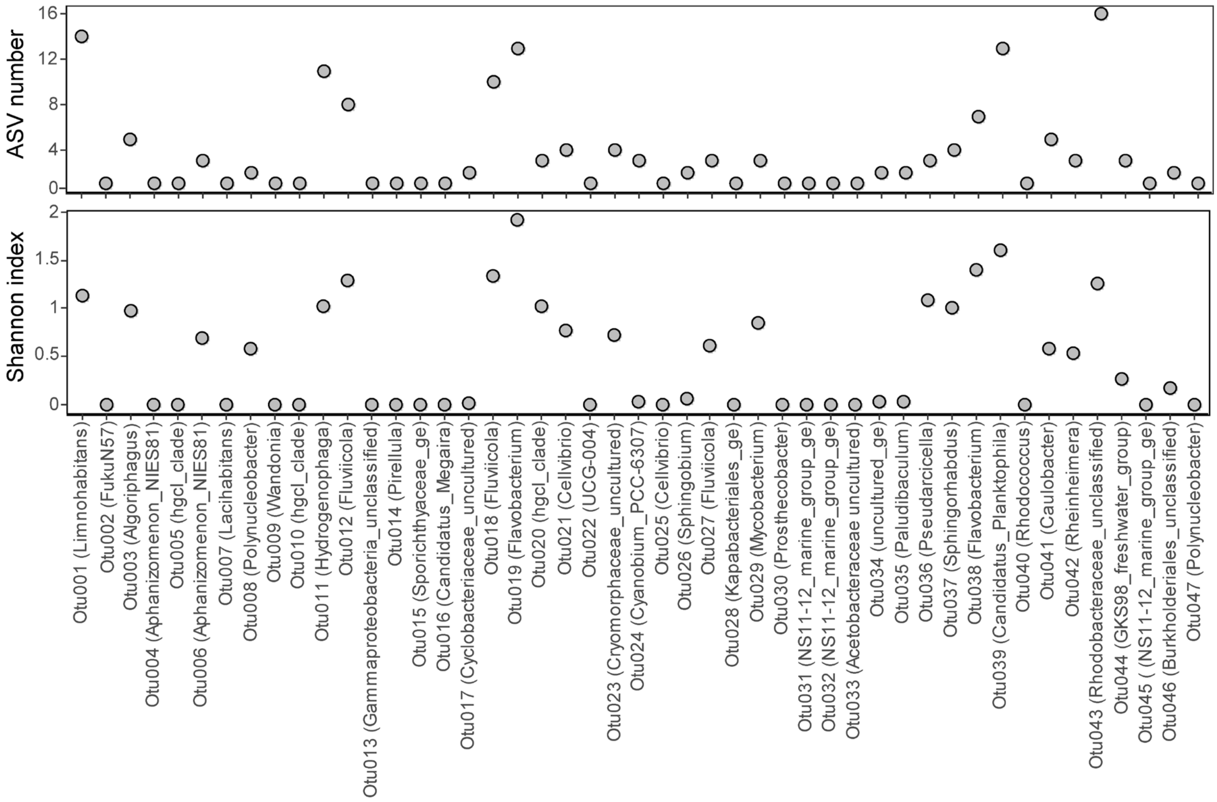

Supplement: Supplementary file 1 [file Presentation_1.pdf]
